# Spatial proteomics: technical evolution and applications in cancer research

**DOI:** 10.3389/fcell.2026.1914317

**Published:** 2026-09-24

**Authors:** Xiaoxiao Tian, Di Huang, Jiaxiang Hu

**Affiliations:** 1 Department of Gastroenterology, The First Affiliated Hospital, College of Clinical Medicine, Henan University of Science and Technology, Luoyang, Henan, China; 2 Department of Child Healthcare, The Third Affiliated Hospital of Zhengzhou University, Zhengzhou, Henan, China; 3 Department of Scientific Research, The First Affiliated Hospital of Zhengzhou University, Zhengzhou, Henan, China

**Keywords:** cancer, mass spectrometry imaging, spatial biomarkers, spatial proteomics, tumor microenvironment

## Abstract

Spatial proteomics is reshaping tissue-based cancer research by linking protein expression to cellular identity, tissue architecture and clinical behavior. Conventional proteomics has provided powerful tools for protein identification and quantification, but most bulk workflows erase the spatial relationships that define tumor ecology. In contrast, spatial proteomics combines multiplexed imaging, mass spectrometry-based tissue analysis, region-selected profiling and computational spatial statistics to measure proteins in their native tissue context. This review summarizes the development of proteomics from early separation and mass spectrometry technologies to current spatial platforms. It then focuses on cancer applications, including tumor heterogeneity, tumor microenvironment, antitumor immunity, immunotherapy response, spatial biomarkers and treatment resistance. Across these areas, spatial proteomics is most valuable when it converts descriptive tissue patterns into testable hypotheses about cell states, neighborhood interactions and therapeutic vulnerabilities. The field now needs standardized workflows, clinically anchored cohorts and functional validation to move from high-dimensional tissue maps to robust translational tools.

## 1 Introduction

Proteins are the principal executors of cellular function and remain among the most widely used biomarkers and therapeutic targets in oncology. Mass spectrometry-based proteomics has transformed biomedical research by enabling large-scale protein identification, quantification and pathway analysis across cells, tissues and biofluids. Conventional bulk proteomics, however, usually requires tissue homogenization. This workflow averages signals from malignant, stromal, vascular and immune compartments, and it removes the architectural context in which proteins act (Palla et al., 2022a). Multimodal spatial studies increasingly show that this context is biologically informative across tissues (Guilliams et al., 2022). This limitation is particularly important in cancer because invasion, immune escape and treatment resistance often emerge from local multicellular interactions rather than from uniform molecular programs (Hsieh et al., 2022).

Spatial proteomics addresses this problem by measuring protein abundance, localization and cellular context in intact or spatially registered tissue. The field builds on spatial molecular biology, multiplexed imaging and mass spectrometry imaging, but its emphasis is distinct. It asks not only whether a protein is expressed, but where it is expressed, which cell type carries the signal and which neighboring cells define its functional context (Mund et al., 2022a). Compared with spatial transcriptomics, spatial proteomics is closer to functional effector biology because proteins execute signaling, metabolism and cell-cell communication. Compared with conventional immunohistochemistry, it offers higher multiplexing and more quantitative spatial readouts from the same tissue section (Digre and Lindskog, 2021).

For oncology, this shift is more than a technical upgrade. Tumors are spatially organized ecosystems in which malignant clones, fibroblasts, endothelial cells, immune cells and extracellular matrix form local niches. Spatial protein measurements can show whether cytotoxic T cells enter tumor nests, whether macrophages accumulate near necrotic regions and whether fibroblasts form barriers that exclude immune cells (Keren et al., 2018). These tissue-level relationships are central to interpreting tumor heterogeneity, immunotherapy response and therapy-induced resistance (Di Mauro and Arbore, 2024). They cannot be reliably inferred from bulk proteomes or dissociated single-cell data alone.

This review summarizes how proteomics evolved from protein separation and peptide identification toward tissue-resolved protein mapping. Technical platforms and computational analysis are discussed briefly to provide methodological context. The major focus is the application of spatial proteomics in cancer research, including tumor heterogeneity, tumor microenvironment, immune response, spatial biomarkers and treatment resistance. The review also highlights a central tension in the field: spatial proteomics generates biologically rich tissue maps, but these maps must be standardized, validated and connected to clinical decisions before they can become routine translational tools (Liu et al., 2024).

## 2 From proteomics to spatial proteomics: a brief developmental trajectory

The evolution from conventional proteomics to spatial proteomics reflects a gradual expansion in what proteomic analysis is expected to resolve: first protein separation, then protein identification, quantitative comparison, subcellular localization and, ultimately, tissue-resolved biological interpretation (Figure 1). This trajectory shows that spatial proteomics did not replace earlier proteomic strategies, but emerged from their convergence with imaging, microdissection, mass spectrometry and computational analysis, enabling protein abundance to be interpreted in relation to cell identity, anatomical structure and local tissue context.

**FIGURE 1:**
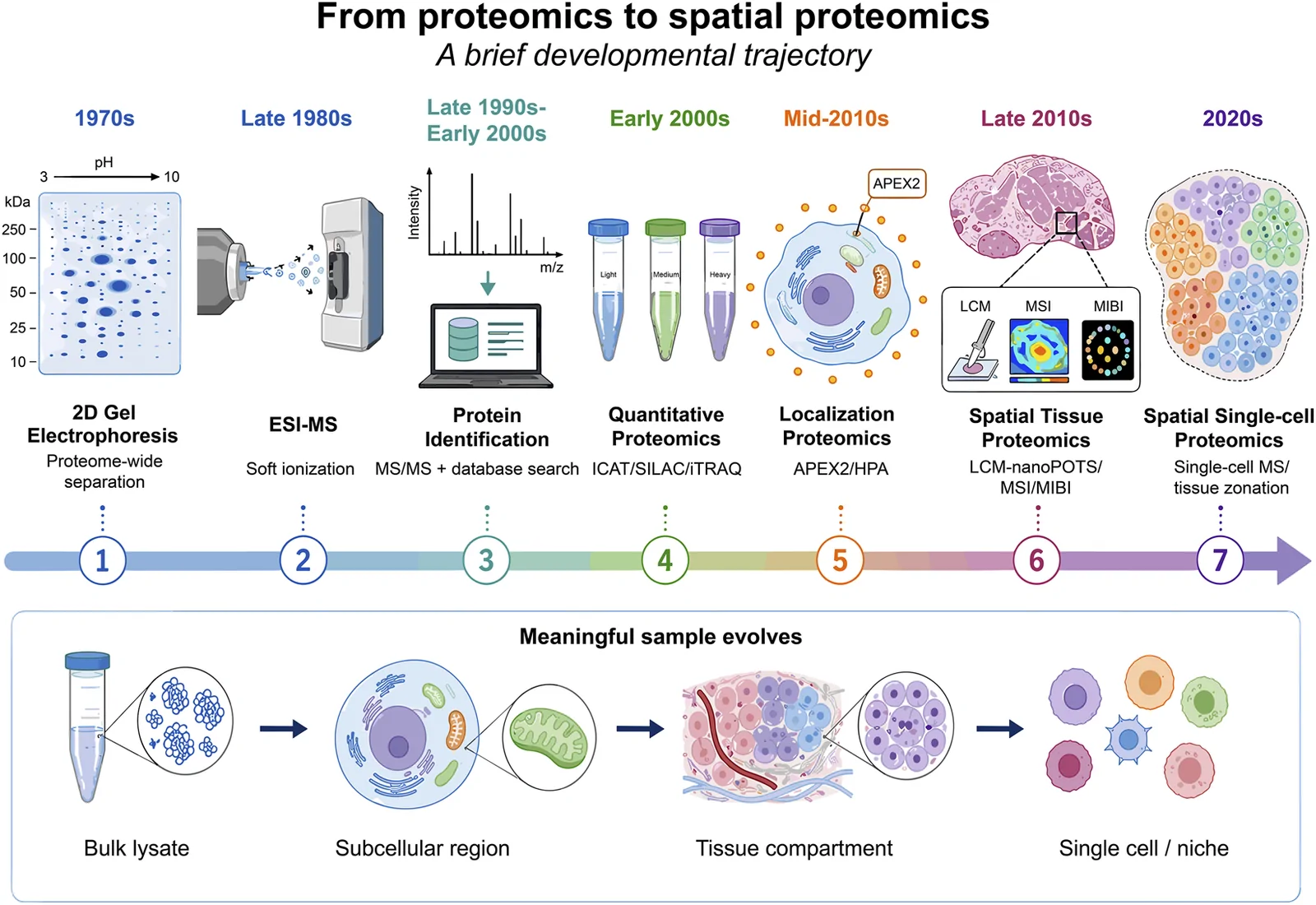
Developmental trajectory from proteomics to spatial proteomics. Evolution of proteomics from bulk protein separation and mass spectrometry-based identification to quantitative, localization, tissue-level and single-cell spatial proteomics. The meaningful analytical unit has shifted from bulk lysates to subcellular regions, tissue compartments and single-cell niches.

In the mid-1970s, proteomics began with the problem of separating complex protein mixtures before proteins could be identified at scale. High-resolution two-dimensional gel electrophoresis, reported in 1975, combined isoelectric focusing with SDS-PAGE and provided an early proteome-wide view of protein expression (O'Farrell, 1975). This method allowed many proteins to be separated and compared across biological states. It also established the idea that a proteome could be mapped systematically, although it favored abundant soluble proteins and had limited reproducibility for difficult targets.

By the late 1980s and early 1990s, the center of protein analysis began to shift from gels to mass spectrometry. Electrospray ionization, introduced for large biomolecules in 1989, made it possible to transfer fragile peptides and proteins into the gas phase without extensive fragmentation (Fenn et al., 1989). This innovation improved sensitivity, increased liquid chromatography compatibility and moved protein analysis toward peptide-centered workflows.

During the 1990s and early 2000s, protein identification became scalable because peptide spectra could be linked to sequence databases. Database-search algorithms enabled tandem mass spectra to be matched with peptide sequences from protein databases (Perkins et al., 1999). Peptide mass fingerprinting provided another route for identifying proteins by matching measured peptide masses to sequence databases (Henzel et al., 1993). Together, these approaches turned mass spectrometry into a high-throughput discovery platform.

From the late 1990s to the mid-2000s, quantitative proteomics became a major turning point. ICAT enabled isotope-coded comparison of complex protein mixtures and made relative quantification more systematic (Gygi et al., 1999). SILAC, introduced in the early 2000s, used metabolic labeling in cell culture and became a robust strategy for comparing cellular proteomes (Ong et al., 2002). iTRAQ then extended multiplexed peptide quantification across several samples and improved throughput for comparative studies (Ross et al., 2004). These technologies made proteomics suitable for comparing biological states and treatment effects, but most workflows still required homogenized samples.

In the 2010s, subcellular and localization-focused proteomics began to recover the missing spatial dimension (Christopher et al., 2021). APEX2-based proximity labeling enabled spatially resolved proteomic mapping in living cells (Hung et al., 2016). Organelle and temporal proteomics showed that protein localization changes across infection, signaling or cellular state transitions (Jean Beltran et al., 2016). LOPIT combined with differential ultracentrifugation further improved high-resolution spatial proteomic mapping (Geladaki et al., 2019). The Human Protein Atlas provided a large-scale spatial map of protein localization across human cells and tissues (Thul and Lindskog, 2018). SubCellBarCode later extended this idea by mapping protein localization and relocalization across the proteome (Orre et al., 2019). These resources made protein position a core biological variable rather than an annotation added after quantification.

From the late 2010s onward, spatial proteomics emerged more clearly as tissue imaging, microdissection, mass spectrometry and computational analysis converged (Lobingier et al., 2017). Laser capture microdissection coupled with automated nanodroplet processing improved proteome measurement from histologically defined regions (Zhu et al., 2018). Automated mass spectrometry imaging mapped thousands of proteins from tissue sections at defined spatial resolution (Piehowski et al., 2020). Multiplexed ion beam imaging showed that high-dimensional protein imaging could define tumor-immune architecture at single-cell resolution. Spatial single-cell mass spectrometry further illustrated how tissue zonation can be measured directly at the proteome level (Rosenberger et al., 2023).

In the 2020s, the unit of proteomic analysis continued to change. Early proteomics treated cells, tissues or biofluids as bulk biochemical material. Quantitative proteomics made state comparisons more reliable, but still treated each sample as one molecular compartment. Spatial proteomics changes this sampling logic. Tissue expansion now offers another route for spatially resolved proteomic measurement (Li et al., 2022). The unit of analysis can now be a cell, a subcellular region, a histological compartment, a tumor border or a multicellular niche. This matters because proteins often exert different functions depending on local concentration, localization and interaction partners (Boisvert et al., 2012).

Viewed chronologically, the field has moved from separation, to identification, to quantification, to localization and finally to tissue-resolved interpretation. Shotgun proteomics and isotope labeling identify and quantify proteins with depth. Spatial platforms ask which molecular signal belongs to which cell and tissue structure. These approaches are complementary rather than competing. Bulk LC-MS/MS provides unbiased coverage, while spatial methods provide anatomical localization and cell-cell context. The most informative studies increasingly combine these strategies. Spatial and cell-type resolved atlases of healthy tissues show how such integration can define baseline tissue organization (Dyring-Andersen et al., 2020).

## 3 Technologies and computational analysis

Current spatial proteomics platforms differ in molecular coverage, spatial resolution and clinical scalability, making technology choice central to the biological question being asked (Figure 2) (Table 1).

**FIGURE 2:**
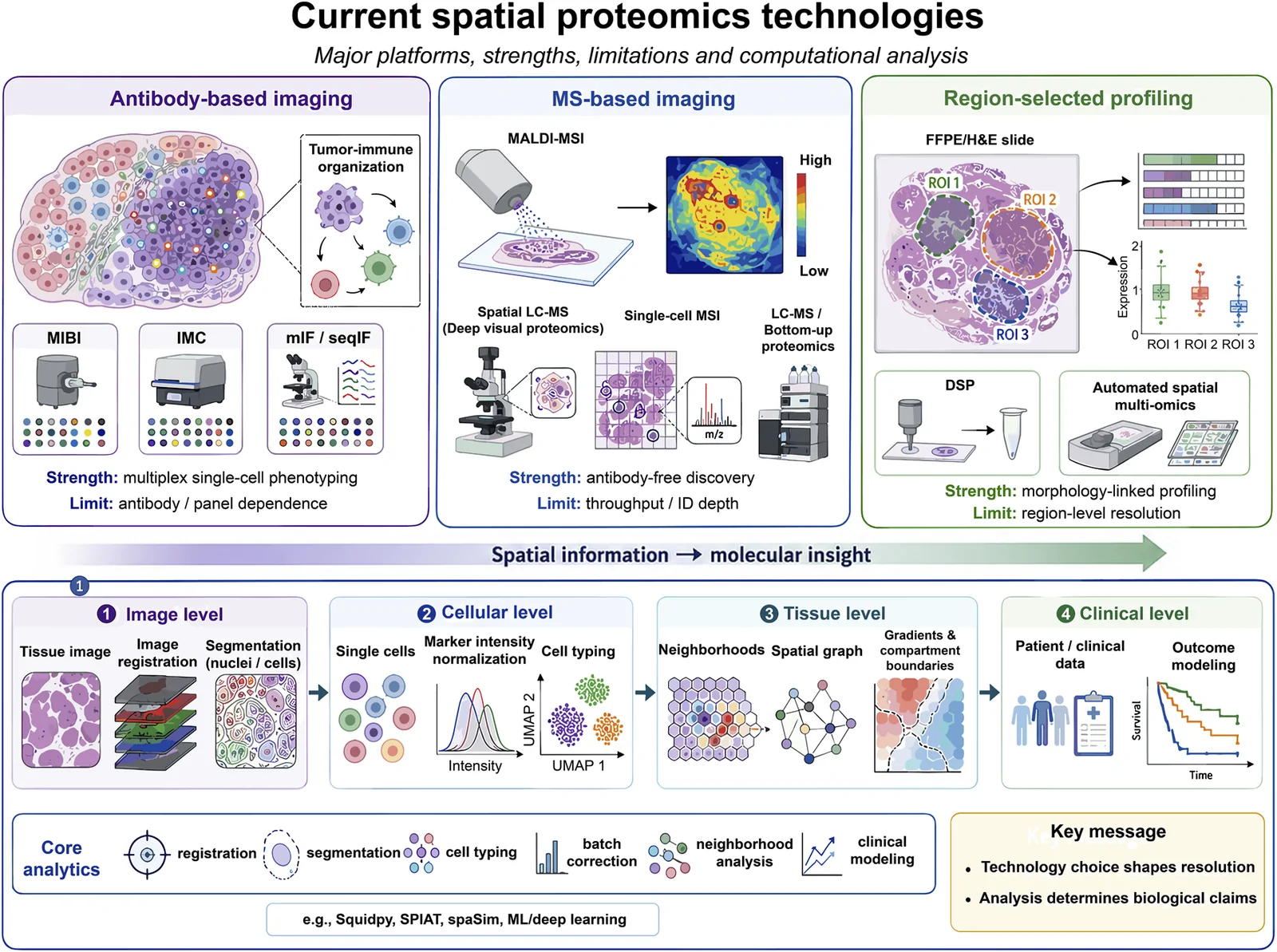
Major technologies and analysis workflow in spatial proteomics. Overview of antibody-based imaging, mass spectrometry-based imaging and region-selected profiling platforms, with their main strengths and limitations. The analytical workflow proceeds from image registration and cell segmentation to cell typing, neighborhood analysis and clinical modeling.

**TABLE 1:** Decision-oriented comparison of spatial proteomics platforms.

Platform	Typical multiplex/coverage	Spatial unit/resolution	FFPE, throughput and antibody dependence	Best-fit question	Main advantage, limitation and translational outlook
IMC	Usually 30–50 markers	∼1 μm pixels; single-cell/subcellular	FFPE compatible; moderate area throughput; metal-tagged antibodies	Which cell states and contacts occur in a defined tissue region?	Low autofluorescence and strong phenotyping; slow laser ablation, specialized instrumentation and panel validation. Moderate translational potential for locked targeted panels
MIBI-TOF	Usually ∼40–50 markers; higher capacity reported	∼0.3–1 μm; single-cell/subcellular	FFPE compatible; moderate-to-low area throughput; metal-tagged antibodies	How are rare cells and nanoscale-to-cellular neighborhoods organized?	High spatial resolution and dynamic range; costly specialized instrumentation and limited routine availability. Moderate translational potential
CODEX/PhenoCycler	Commonly 40–100+ markers	Optical, single-cell/subcellular	FFPE compatible with validated panels; moderate-to-high sample throughput; DNA-barcoded antibodies	How do immune and stromal neighborhoods vary across large cohorts?	High plex and broad tissue coverage; repeated cycles, registration and antibody validation add complexity. High potential for targeted translational studies
t-CyCIF	Up to ∼60 markers in published implementations	Optical, single-cell/subcellular; whole-slide capable	FFPE compatible; parallel slide processing but many cycles; conventional antibodies	Can whole-slide architecture and signaling states be screened accessibly?	Uses conventional microscopes and flexible panels; cycle time, tissue loss and order effects require controls. High potential after automation and panel locking
GeoMx DSP	Up to ∼570 antibodies (Immuno-Oncology Proteome Atlas); whole transcriptome (∼18,000 genes) in parallel; Discovery Proteome Atlas supports >1,200 protein targets	Pathologist-selected ROIs, usually multicellular rather than intrinsically single-cell	Strong FFPE compatibility and cohort scalability; antibody/oligonucleotide affinity reagents	Which histological compartments carry outcome-associated signals? (high-plex protein and transcript co-profiling)	Clinical-sample friendly and pathology guided; ROI averaging and selection bias limit cellular inference. High-plex protein panels (IPA: ∼570 targets; DPA: >1,200 targets) now available. Relatively high near-term translational potential
MALDI-MSI/spatial LC-MS	Untargeted hundreds-to-thousands of molecular features or proteins, workflow dependent	Typically ∼10–100 μm; cell-scale methods are emerging	Fresh-frozen is common; FFPE requires optimized preparation; moderate throughput; antibody independent for discovery	Which unexpected molecules or protein programs map to histology?	Unbiased discovery and molecular breadth; identification, quantification, sensitivity and spatial-resolution trade-offs. Moderate translational potential after assay simplification
SIMS/nanoSIMS/DESI-MSI	Chemical/isotopic features; protein specificity is limited unless targeted labels are used	Sub-micrometre to nanoscale for SIMS; tens-to-hundreds of micrometres for DESI	Tissue compatible but specialized workflows; generally antibody independent, except targeted immunolabeling	Where are subcellular chemical or isotope-tracer patterns, or ambient-surface molecular signals?	Distinct chemical resolution or minimal-preparation imaging; limited protein identification, destructive sampling or low throughput constrain routine clinical use
DBiT-seq/spatial-CITE-seq	∼20–273 antibody-derived protein targets plus transcriptome in published studies	∼10–25 μm spatial pixels	Fixed tissue; moderate throughput; DNA-tagged antibodies plus microfluidics and sequencing	Which protein and transcript programs co-occur in the same spatial grid?	Exceptional multi-omic plex; pixel mixing, sequencing burden and specialized handling remain barriers. Emerging translational potential
AI virtual spatial proteomics	Model-specific; HEX predicts 40 proteins	Computational patches or inferred cell/neighborhood maps	Scales to archived H&E/FFPE slides; no antibody at inference, but measured labels are required for training	Can measured spatial biomarkers be screened or triaged at pathology scale?	Low incremental tissue use and high scalability; domain shift, calibration and non-equivalence to measured protein remain major risks. Promising as a complement, not a replacement

Current spatial proteomics technologies can be grouped into antibody-based imaging, mass spectrometry-based imaging and region-selected molecular profiling. Antibody-based platforms such as multiplexed ion beam imaging, imaging mass cytometry and multiplexed immunofluorescence quantify many protein markers with single-cell spatial resolution. Multiplexed ion beam imaging revealed structured tumor-immune organization in triple-negative breast cancer (Angelo et al., 2014). Imaging mass cytometry enabled highly multiplexed subcellular protein imaging and was subsequently extended to simultaneous multiplexed imaging of mRNA and proteins in breast cancer tissue (Giesen et al., 2014; Schulz et al., 2018). The strength of these methods is cellular phenotyping in intact tissue, whereas their limitation is dependence on antibody quality and panel design. Sequential immunofluorescence workflows show how hyperplex imaging can expand antibody-based spatial proteomics (Rivest et al., 2023).

Cyclic antibody imaging is a major subclass within this group. CODEX, commercialized as PhenoCycler, uses DNA-barcoded antibodies and iterative fluorescent readout to generate high-plex single-cell maps, whereas tissue-based cyclic immunofluorescence (t-CyCIF) repeats staining, imaging and fluorophore inactivation on conventional optical microscopes (Goltsev et al., 2018; Lin et al., 2018). These modalities are directly relevant to the application literature summarized below: PhenoCycler-Fusion was used in the NSCLC and head-and-neck cohorts described by Aung et al. and Sadeghirad et al., and cyclic imaging supported the three-dimensional colorectal cancer atlas reported by Lin et al. Their principal strengths are broad tissue coverage and cellular phenotyping; registration error, cycle-dependent antigenicity, tissue loss and antibody-panel validation are the main constraints.

Mass spectrometry-based approaches offer a different balance of strengths. MALDI-MSI and spatially resolved LC-MS workflows are less dependent on predefined antibodies and can discover unexpected molecular species (Gustafsson et al., 2011). Deep visual proteomics links image-guided cell selection with high-depth mass spectrometry and thereby connects morphology with proteome states. MALDI-guided spatial proteomics can combine targeted histological information with untargeted molecular discovery (Claes et al., 2023). Recent bottom-up spatial proteomics protocols are moving toward single-cell resolution, although throughput and protein identification depth remain important constraints (Grgic et al., 2024). Single-cell mass spectrometry imaging with integrated microscopy further extends this direction (Potthoff et al., 2025).

Additional mass spectrometry imaging modalities occupy distinct analytical niches. Secondary ion mass spectrometry (SIMS) and nanoSIMS provide submicrometre or nanoscale chemical and isotope maps, with targeted protein visualization possible through isotope- or nanoparticle-labelled affinity reagents. Desorption electrospray ionization mass spectrometry imaging (DESI-MSI) samples tissue surfaces under ambient conditions with limited preparation (Passarelli and Winograd, 2011; Takáts et al., 2004). These approaches extend spatial molecular analysis beyond MALDI, but destructive sampling, restricted protein identification or lower throughput currently limit routine clinical use.

Region-selected spatial profiling occupies an intermediate position between imaging and global proteomics. Automated spatial multi-omics platforms increase throughput for region-linked molecular profiling. Digital spatial profiling and related approaches allow investigators to select regions of interest based on morphology, marker expression or pathology review (Merritt et al., 2020). These regions can then be linked to protein or transcript measurements. This design is useful for formalin-fixed paraffin-embedded clinical material because it connects molecular readouts to pathologist-defined compartments (Vickovic et al., 2022). Its limitation is that the spatial unit is often a selected region rather than a fully resolved single cell.

Sequencing-based deterministic barcoding adds a fourth branch to the taxonomy. DBiT-seq co-maps mRNAs and antibody-derived protein tags on spatial grids, while spatial-CITE-seq extends protein multiplexing to hundreds of epitopes together with the transcriptome (Liu et al., 2023; Liu et al., 2020). These methods provide exceptional multi-omic breadth but remain antibody dependent for protein measurement and trade direct subcellular imaging for barcoded pixels, microfluidic handling and sequencing.

Platform selection should therefore begin with the endpoint rather than the highest marker count. IMC or MIBI is well suited to high-resolution cell-state and contact questions in a limited tissue area; CODEX/PhenoCycler or t-CyCIF is preferable when whole-slide or large-cohort cellular architecture is central; GeoMx DSP is efficient for pathology-defined FFPE compartments and clinically annotated cohorts; and MALDI-MSI or spatial LC-MS is preferred for antibody-independent discovery. Deterministic barcoding is most useful when protein-transcript co-mapping is essential, whereas virtual spatial proteomics is a scalable inference layer for archived H&E material. For GeoMx DSP, the platform has evolved substantially beyond its original design; it now supports up to approximately 570 antibodies through the Immuno-Oncology Proteome Atlas (IPA) and over 1,200 protein targets through the Discovery Proteome Atlas (DPA), both run in parallel with whole-transcriptome profiling (∼18,000 genes) (Lubo et al., 2024). For near-term clinical translation, targeted, FFPE-compatible and automatable platforms have the clearest path, but only after marker panels, acquisition settings and analysis pipelines are locked and externally validated.

Computational analysis determines whether spatial proteomic data become biological insight. Image registration, segmentation, cell typing, batch correction, neighborhood analysis and clinical modeling are required before spatial signals can be interpreted. Squidpy provides a scalable framework for spatial omics analysis and supports neighborhood and spatial graph analysis (Palla et al., 2022b). SPIAT and spaSim were developed to characterize and simulate tissue microenvironments from spatial data (Feng et al., 2023). Newer machine learning models improve cell annotation and segmentation, but algorithmic choices can still reshape biological conclusions (Shaban et al., 2024). Deep learning reviews of highly multiplexed tissue imaging emphasize similar issues for segmentation and feature extraction (Zidane et al., 2023).

Spatial proteomics requires analysis at several levels. At the image level, nuclei and cell boundaries must be segmented accurately. Segmentation errors propagate to cell-type proportions, distance metrics and apparent cell-cell contacts, so performance should be audited against expert annotations across representative tissues. At the cellular level, marker intensity must be normalized and translated into cell states. Autofluorescence, spillover, thresholding and batch correction can merge or split phenotypes and destabilize biomarker selection. At the tissue level, neighborhoods, gradients and compartment boundaries must be defined. ROI sampling, neighborhood scale and null-model choice can create spurious enrichment or obscure rare niches. At the clinical level, these spatial features must be linked to outcomes without overfitting. Small cohorts, center effects, data leakage and uncalibrated models can inflate apparent prognostic or stratification performance. This hierarchy explains why computational methods are not technical details. Reliable spatial proteomics also depends on data-analysis foundations that can control spatial and quantitative variability (Gatto et al., 2014). They determine which biological claims can be made from the data (Chen et al., 2024).

A computational pipeline is clinically fit for purpose when it is prespecified or locked before endpoint evaluation, versioned and reproducible from raw data, tested with positive and negative controls, robust to reasonable segmentation and neighborhood parameters, externally validated across centers or platforms, calibrated for the intended endpoint, and accompanied by uncertainty and failure analyses. Under these conditions, computation can convert images into stable cell-state and neighborhood features, control technical variation and support patient-level models that add information beyond established clinicopathological covariates. Without these safeguards, a spatial signature may encode scanner, batch or ROI-selection effects rather than tumor biology (Samorodnitsky and Wu, 2024; Schapiro et al., 2022).

## 4 Advantages of spatial proteomics

The distinctive value of spatial proteomics is not simply higher multiplexing. It is the ability to interpret protein expression within preserved tissue architecture. Bulk proteomics can identify altered pathways, but it cannot distinguish whether a signal arises from malignant cells, immune infiltrates, fibroblasts, endothelial cells or rare cellular niches (Figure 3). Early spatial tissue proteomics in hepatocellular carcinoma already showed that regional protein measurements could quantify intertumor and intratumor heterogeneity more directly than averaged tissue profiling (Buczak et al., 2018). Deep visual proteomics further extended this logic by linking cellular morphology, artificial intelligence-guided microdissection and ultrasensitive mass spectrometry to single-cell-resolved protein states (Mund et al., 2022b). More recent low-input spatial tissue proteomics workflows also show that protein-level analysis is becoming feasible in smaller and more precisely defined tissue regions (Makhmut et al., 2023).

**FIGURE 3:**
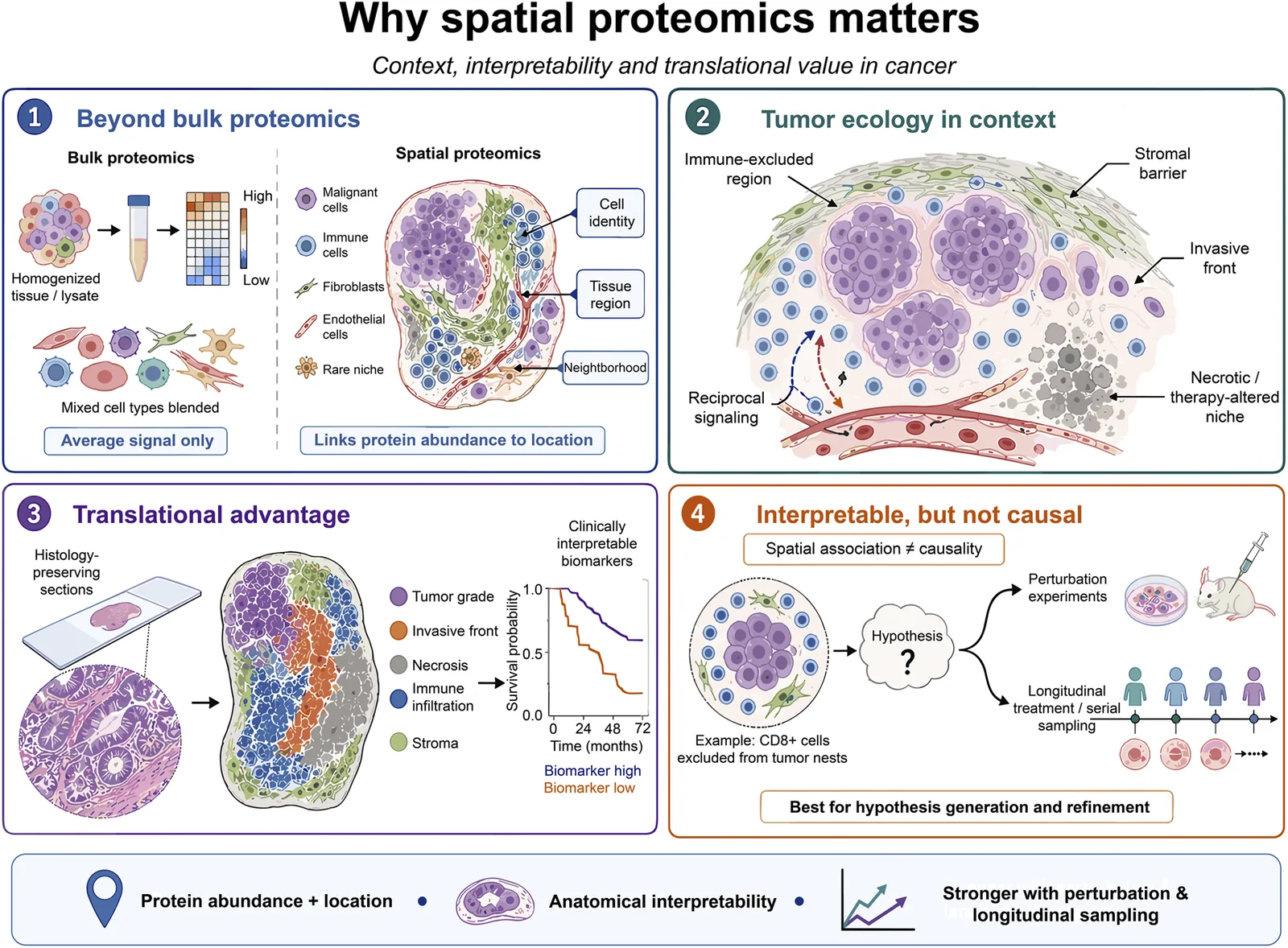
Advantages of spatial proteomics in cancer research. Spatial proteomics links protein abundance with tissue location, cellular identity and neighborhood structure. Its main value lies in interpreting tumor ecology, identifying clinically meaningful spatial biomarkers and generating hypotheses for perturbation or longitudinal validation.

This spatial context is particularly critical in cancer. Tumors are ecological systems composed of malignant clones, immune cells, fibroblasts, endothelial cells and extracellular matrix. Their behavior depends on adjacency, exclusion, invasion and reciprocal signaling. Similar single-cell spatial imaging of ductal carcinoma *in situ* and invasive breast cancer revealed progressive stromal remodeling during invasion (Risom et al., 2022). Spatially resolved multi-omics in glioblastoma further demonstrated bidirectional tumor-host dependencies that would be difficult to infer from dissociated or bulk profiles (Ravi et al., 2022). Integrative spatial analysis of glioblastoma and 3D mapping of colorectal cancer also showed that tissue architecture can reveal layered tumor organization and state transitions across local microenvironments (Greenwald et al., 2024; Lin et al., 2023).

Spatial proteomics also provides a practical advantage for translational studies because it can be applied to tissue sections that preserve histology. This allows molecular analysis to be aligned with tumor grade, invasive fronts, necrosis, immune infiltration, vascular structures and stromal compartments. The Human Protein Atlas illustrates the broader value of mapping proteins in tissue context at scale. In glioblastoma, spatial proteome analysis identified molecular signatures and survival-associated markers, showing how tissue-resolved protein measurements can be connected to clinical outcome (Duhamel et al., 2022). AI-enabled virtual spatial proteomics from histopathology also suggests that spatial protein information may eventually be scaled through computational links between morphology and molecular state (Li et al., 2026).

AI-based virtual spatial proteomics is a computational extension rather than a direct measurement platform. Models such as H&E-to-protein expression (HEX) learn mappings from routine histology to matched high-plex protein images and generate virtual marker maps at scale; in six independent NSCLC cohorts, such maps supported prognostic and immunotherapy-response modeling (Li et al., 2026). More broadly, multiscale oncology AI can link radiology, whole-slide morphology and spatial molecular data across macroscopic and microscopic scales (Tarhini and El Naqa, 2025). Its translational appeal is low incremental tissue use and compatibility with archived H&E slides. However, predicted expression is sensitive to staining and scanner shifts, cohort composition and training-label quality. Virtual maps therefore require protein-level calibration, external multi-institutional validation and prospective endpoint testing, and should be treated as inferred biomarkers rather than substitutes for measured spatial proteomics.

Another advantage is interpretability. A spatial protein feature can often be described in anatomical terms, such as CD8-positive cells excluded from tumor nests, macrophages enriched near invasive fronts or fibroblasts forming stromal barriers. These features are easier for clinicians and pathologists to interpret than abstract latent variables from bulk omics. Conserved spatial subtypes and cellular neighborhoods of cancer-associated fibroblasts provide a clear example of how protein and cell-state information can be translated into tissue-level biological concepts (Liu et al., 2025). This interpretability may improve clinical adoption, provided that spatial metrics are standardized and reproducible. Standardized workflows for histology-compatible spatial proteomics therefore represent an important step toward cross-cohort comparison and clinical translation (Nordmann et al., 2023).

The field should nonetheless avoid overclaiming. Spatial association is not causality. A cell neighborhood can nominate mechanisms, but mechanistic claims require orthogonal measurement and perturbation in models that preserve the relevant cell-cell context. Counterfactual learning from large-scale spatial proteomic profiles further illustrates how spatial maps can nominate perturbations, although such predictions still require experimental validation. In this sense, spatial proteomics is best viewed as a hypothesis-generating and hypothesis-refining approach rather than a standalone proof of mechanism.

## 5 Applications in cancer research

In cancer research, spatial proteomics is most informative when it connects protein expression to the physical organization of tumor tissue. This section therefore focuses on studies that measured proteins, peptides or protein-defined cell states in spatially resolved tissue contexts, including multiplexed antibody imaging, digital spatial profiling, mass spectrometry imaging, imaging-guided microdissection and deep visual proteomics (Table 2). Evidence is compared below by platform, cohort design, cross-study convergence and proximity to a clinically testable endpoint rather than by novelty alone.

**TABLE 2:** Representative cancer studies using spatial proteomics technologies.

Applications in cancer research	Tumor type	Sample type/cohort	Spatial proteomics platform	Main spatial-proteomic contribution	References
Tumor heterogeneity	Hepatocellular carcinoma	HCC tumor regions	Spatial tissue proteomics/regional LC-MS-based proteomics	Quantified intertumor and intratumor proteomic heterogeneity	(Buczak et al., 2018)
	Pancreatic tumor	FFPE pancreatic tumor sections	SCPro: mIHC-guided LMD + iPAC LC-MS/MS	Resolved malignant and stromal protein states in local tissue regions	(Xu et al., 2024)
	Colorectal adenoma	Human colorectal adenoma tissue	Deep visual proteomics	Stratified precursor lesions by spatial molecular features	(Kabatnik et al., 2024)
	Colorectal cancer	Patient-derived CRC samples	Functional and spatial proteomics	Mapped regional intra- and intercellular signaling crosstalk	(Plattner et al., 2023)
	Diffuse malignant mesothelioma	Mesothelioma tissue regions	FTIR imaging-guided LCM proteomics	Linked histological heterogeneity to regional molecular differences	(Großerueschkamp et al., 2017)
Tumor microenvironment	Hepatocellular carcinoma	HCC tissue sections	Pathology-guided spatial proteomics	Identified stromal architectures and CAF subpopulations with opposing outcome associations	(Cheng et al., 2025)
	Pan-cancer CAF analysis	Multiple tumor and tissue contexts	Spatial transcriptomic and proteomic landscape analysis	Placed CAF superclusters in spatial proteomic and tissue-context maps	(Foster et al., 2022)
	Chordoma	Chordoma tissue sections	GeoMx DSP, DIA proteomics and multiplexed quantitative immunofluorescence	Linked ER-stress CAFs to GMFG-ITGB1 signaling and immune remodeling	(Zhang et al., 2024b)
	High-grade serous ovarian cancer	BRCA1/2-mutated and non-mutated HGSOC tumors	Highly multiplexed immunofluorescence imaging	Defined single-cell tumor-immune microenvironment features associated with BRCA status	(Launonen et al., 2022)
	Colorectal cancer	CRC tissue and CAF analyses	Spatial proteomics with single-cell analysis	Identified CAF-mediated effector T-cell inhibition through NECTIN2 signaling	(Agorku et al., 2024)
Immunity and immunotherapy	Primary melanoma	Primary melanoma tissue specimens	Single-cell multiplexed imaging	Showed functional heterogeneity of lymphocytic infiltration patterns	(Bosisio et al., 2020)
	Metastatic melanoma treated with TIL-ACT	Longitudinal tumor samples from 13 patients	Spatial proteomic analysis	Associated response with pre-existing CD8 T-cell-myeloid networks	(Barras et al., 2024)
	Advanced NSCLC	Patients treated with bispecific antibody	GeoMx DSP/spatial multi-omics	Linked tumor ecosystem heterogeneity to immunotherapy efficacy	(Song et al., 2023)
	Advanced NSCLC	234 advanced NSCLC patients across cohorts	PhenoCycler-Fusion spatial proteomics plus GeoMx DSP WTA/CTA	Developed spatial signatures for predicting immunotherapy outcomes	(Aung et al., 2025)
	Muscle-invasive bladder cancer	Neoadjuvant chemoimmunotherapy cohort	GeoMx DSP/spatial proteomic profiling	Associated spatial TME relationships with pathologic response	(Beckabir et al., 2024)
​	Head and neck cancer	Recurrent/metastatic HNSCC cohorts	GeoMx DSP and Akoya PhenoCycler-Fusion	Linked tertiary lymphoid aggregate dynamics to immunotherapy response	(Sadeghirad et al., 2024)
	Hepatocellular carcinoma	Clinical trial tissue samples	Imaging mass cytometry spatial multiomics	Supported immunotherapy response prediction and biomarker discovery	(Zhang et al., 2024a)
Spatial biomarkers	Triple-negative breast cancer	TNBC TMA specimens	NanoString GeoMx DSP	Identified spatial prognostic features of adjuvant therapy response	(Kulasinghe et al., 2021)
	Colorectal cancer metastasis	Colon tumor and metastasis tissue regions	NanoString GeoMx DSP	Identified spatial proteomic signatures of colon tumor metastasis	(Levy et al., 2023)
	Head and neck squamous cell carcinoma	Primary and recurrent HPV-negative HNSCC tumors	Single-cell spatial proteomics	Associated tumor-immune spatial architecture with clinical outcome	(Blise et al., 2022)
	Prostate cancer	MRI-visible and MRI-invisible clinically significant prostate cancer	Multiplex IF/IHC spatial proteomics	Connected mpMRI heterogeneity to spatial protein and cellular features	(Pachynski et al., 2021)
	Clear cell renal cell carcinoma	ccRCC tissue specimens	MALDI imaging mass spectrometry	Mapped heterogeneous renal cancer proteome features	(Morgan et al., 2013)
	Papillary thyroid carcinoma	Papillary thyroid carcinoma tissue sections	MALDI-TOF imaging mass spectrometry	Identified and validated candidate biomarker proteins	(Min et al., 2014)
	Bladder cancer	Bladder carcinoma *in situ* and reactive atypia regions	FTIR imaging-guided proteomics	Identified a candidate histochemical biomarker for diagnostic discrimination	(Witzke et al., 2019)
Drug response and resistance	Head and neck oropharyngeal SCC	Deeply profiled OPSCC cases	Deep spatial-omics with spatial proteomic component	Provided alternative therapeutic targets and rationale for treatment failure	(Causer et al., 2023)
	HER2-positive breast cancer	Longitudinal neoadjuvant therapy samples	Multiplex spatial proteomics	Connected treatment-linked spatial protein features with pathological response	(McNamara et al., 2021)
	Metastatic melanoma and colorectal cancer	Large-scale spatial proteomic profiles	40-plex imaging mass cytometry	Identified perturbations predicted to increase T-cell infiltration	(Wang et al., 2025)

### 5.1 Intratumoral heterogeneity and regional tumor states

Intratumoral heterogeneity is a central application of spatial proteomics because protein programs are often localized to specific tumor regions rather than uniformly distributed across a lesion. Early spatial tissue proteomics in hepatocellular carcinoma showed that regional proteome measurements could quantify both intertumor and intratumor heterogeneity, supporting the need for spatially guided sampling in solid tumors (Buczak et al., 2018). In pancreatic cancer, multimodal single-cell-resolved spatial proteomics combined antibody-guided cell typing with mass spectrometry and revealed malignant and stromal protein programs that varied across local tissue regions (Xu et al., 2024). Deep visual proteomics in colorectal adenomas further showed that morphology-guided spatial proteome measurements can stratify precursor lesions by molecular features beyond routine histopathology (Kabatnik et al., 2024).

Spatial proteomics also helps distinguish regional signaling states from average tumor phenotypes. Functional and spatial proteomic profiling in colorectal cancer showed that intra- and intercellular signaling crosstalk can be organized regionally, indicating that pathway activity should be interpreted in its tissue context (Plattner et al., 2023). Label-free FTIR imaging integrated with laser capture microdissection and proteomics demonstrated a similar principle in diffuse malignant mesothelioma, where histological heterogeneity could be connected to region-specific molecular differences (Großerueschkamp et al., 2017). Together, these studies show that spatial heterogeneity is not simply molecular noise. It is a structured feature of tumor biology that can define malignant states, stromal interfaces and clinically relevant tissue regions.

Across these studies, regional heterogeneity is the most reproducible conclusion: it appears in antibody-guided single-cell imaging, histology-guided microdissection and label-free imaging-proteomics workflows. The exact protein programs are less consistently replicated because tumor types, sampled regions and analytical depth differ. Thus, spatially guided sampling is a robust methodological recommendation, whereas individual regional signatures remain candidates until tested in larger multi-region cohorts.

### 5.2 Tumor microenvironment and stromal organization

The tumor microenvironment is inherently spatial, and spatial proteomics is well suited to study cell-cell proximity, stromal barriers and local paracrine circuits. Spatial proteomic analysis of hepatocellular carcinoma identified stromal architectures and fibroblast subpopulations with opposing associations with clinical outcome, emphasizing that cancer-associated fibroblasts should not be treated as a single uniform compartment (Cheng et al., 2025). Multiomic analysis of cancer-associated fibroblasts further placed CAF states within spatial transcriptomic and proteomic landscapes, supporting the view that fibroblast phenotypes depend on both intrinsic regulatory programs and tissue context (Foster et al., 2022). In chordoma, integration of GeoMx Digital Spatial Profiler, spatial transcriptomics, proteomics and multiplexed immunofluorescence linked hypoxia-induced ER-stress CAFs to paracrine GMFG-ITGB1 signaling and immune remodeling (Zhang T. L. et al., 2024).

Spatial proteomics also broadens microenvironment analysis beyond fibroblasts. Highly multiplexed imaging of high-grade serous ovarian cancer characterized tumor-immune composition at single-cell resolution and revealed immune microenvironment features associated with BRCA1/2 status (Launonen et al., 2022). In colorectal cancer, spatial proteomics combined with single-cell analysis implicated CAF-mediated inhibition of effector T cells through NECTIN2 signaling, providing a mechanistic example of stromal control over antitumor immunity (Agorku et al., 2024). Together, these studies show that spatial proteomics can place cell states, protein markers and signaling axes within local neighborhoods rather than treating the tumor microenvironment as a dissociated cell mixture.

The cross-study signal is strongest at the level of stromal organization: multiple platforms converge on the existence of spatially distinct CAF and immune states. By contrast, specific signaling axes such as NECTIN2 or GMFG-ITGB1 are supported mainly by individual tumor-type studies and require independent perturbational replication. This distinction separates a robust organizing principle from mechanism-specific claims.

### 5.3 Tumor immunity and immunotherapy response

Spatial proteomics has become especially important in tumor immunology because immune response depends on cell location as much as cell abundance. Single-cell multiplexing of primary melanoma showed that lymphocytic patterns are functionally heterogeneous, challenging the assumption that immune infiltration can be interpreted only by density or morphology (Bosisio et al., 2020). In metastatic melanoma treated with tumor-infiltrating lymphocyte adoptive therapy, spatial proteomic analyses showed that response was associated with pre-existing CD8-positive T-cell and myeloid-cell networks, suggesting that baseline immune architecture can influence therapeutic efficacy (Barras et al., 2024).

Non-small-cell lung cancer provides a strong translational example. Spatial multi-omics in advanced NSCLC treated with a bispecific antibody showed that tumor ecosystem heterogeneity influenced immunotherapy efficacy (Song et al., 2023). A larger spatial multi-omics study in advanced NSCLC further developed spatial signatures for predicting immunotherapy outcomes, integrating spatial proteomic and transcriptomic information across clinically annotated cohorts (Aung et al., 2025). In muscle-invasive bladder cancer, spatial relationships in the tumor microenvironment were associated with pathologic response to neoadjuvant chemoimmunotherapy, indicating that treatment response may depend on spatial immune organization rather than marker expression alone (Beckabir et al., 2024). Spatial profiling of tertiary lymphoid aggregates in head and neck cancer similarly linked immune tissue structures to immunotherapy response (Sadeghirad et al., 2024).

The translational promise is clear but should be interpreted carefully. Spatial immune biomarkers are biologically plausible because checkpoint therapy depends on antigen presentation, cell-cell contact, local suppression and stromal exclusion. Clinical-trial spatial multiomics in hepatocellular carcinoma showed that imaging mass cytometry data can be integrated with virtual clinical trials for response prediction and biomarker discovery (Zhang S. et al., 2024). However, spatial immune signatures must still be validated across platforms, cohorts and treatment regimens before they can be used as routine clinical tests.

Among the application areas, treatment-anchored immune studies currently provide the clearest translational evidence because several cohorts link baseline or on-treatment architecture to response. Nevertheless, the reported signatures differ in platform, therapy, tissue sampling and endpoint, and most have not been prospectively locked. The robust conclusion is that spatial organization adds information beyond cell abundance; no universal immune spatial classifier is yet established.

### 5.4 Diagnosis, prognosis and spatial biomarkers

Spatial proteomics changes biomarker discovery by adding location, cellular source and tissue neighborhood to protein abundance. In triple-negative breast cancer, digital spatial profiling identified prognostic features associated with response to adjuvant therapy, showing that compartment-aware protein measurements can refine risk interpretation (Kulasinghe et al., 2021). Digital spatial profiling of colon tumor metastasis identified spatial proteomic signatures associated with metastatic behavior, further supporting the value of tumor- and microenvironment-specific protein measurements (Levy et al., 2023). In head and neck squamous cell carcinoma, single-cell spatial proteomic analysis linked tumor-immune architecture to clinical outcome, suggesting that cellular arrangement can carry prognostic information beyond cell frequency (Blise et al., 2022).

Spatial biomarker discovery is not limited to antibody-based imaging. In prostate cancer, single-cell spatial proteomics helped explain why some clinically significant tumors are invisible on multiparametric MRI, connecting radiographic heterogeneity to molecular and cellular tissue organization (Pachynski et al., 2021). Imaging mass spectrometry mapped heterogeneous protein features in clear cell renal cell carcinoma and identified candidate biomarker proteins in papillary thyroid carcinoma (Min et al., 2014; Morgan et al., 2013). Integrated FTIR imaging and proteomics also identified a candidate histochemical biomarker in bladder cancer, illustrating how label-free imaging and proteomics can support diagnostic discrimination (Witzke et al., 2019). These examples show that spatial biomarkers are most useful when they improve interpretation beyond conventional stage, grade, mutation status or single-marker immunohistochemistry.

Cellular niches are candidate spatial biomarkers defined by the composition, state and geometry of neighboring cells. They can compress high-dimensional maps into interpretable measures of immune exclusion, stromal support or tumor-immune engagement and may stratify response when cell abundance alone does not. Their advantages are biological context, compatibility with serial tissue assessment and potential alignment with pathology. Their limitations are dependence on segmentation, cell-state definitions, neighborhood radius or graph construction, ROI sampling and cohort-specific cutoffs.

Direct head-to-head evidence does not yet support a universal ranking of cellular niches *versus* tertiary lymphoid structures or aggregates for treatment-response prediction. Tertiary lymphoid structures are discrete, pathologist-recognizable immune organizations and may be easier to standardize, whereas data-driven niches capture a broader range of myeloid, T-cell, tumor and stromal arrangements but carry a higher overfitting risk. Comparative studies should evaluate both feature classes in the same cohort using discrimination, calibration, decision-curve benefit and external validation, and should test whether either adds value beyond PD-L1, stage and other established covariates.

### 5.5 Therapeutic targets, drug response and resistance

Therapeutic resistance often emerges in spatially protected niches rather than uniformly throughout a tumor. Spatial proteomics can therefore help identify where residual cells persist, which neighboring cells support them and which protein programs are enriched after therapy. Deep spatial-omics analysis of head and neck carcinomas provided alternative therapeutic targets and a tissue-level rationale for treatment failure, illustrating how spatially resolved profiling can generate clinically interpretable hypotheses (Causer et al., 2023). Longitudinal spatial proteomic characterization of HER2-positive breast tumors during neoadjuvant therapy showed that treatment-linked tissue sampling can connect spatial protein features to pathological response (McNamara et al., 2021).

Computational use of spatial proteomic data is also expanding. A counterfactual learning framework trained on large-scale spatial proteomic profiles identified perturbations predicted to boost T-cell infiltration into tumors, treating spatial proteomics as a design space for therapeutic intervention (Wang et al., 2025). This direction is conceptually powerful, but computationally nominated perturbations still require experimental validation.

The evidence for resistance mechanisms is therefore heterogeneous: longitudinal treatment-linked tissue studies are closer to clinical validation, whereas counterfactual predictions and single-cohort target nominations remain hypothesis generating. Priority should be given to signals that recur across time points, platforms or independent cohorts and can be functionally perturbed.

## 6 Challenges and future perspectives

Several challenges limit the clinical adoption of spatial proteomics. Antibody specificity, tissue processing, segmentation quality and batch effects can strongly influence results. Spatial omics reviews have emphasized that multimodal tissue biology requires careful control of experimental and computational variation. Single-cell and subcellular spatial technologies face additional trade-offs among resolution, molecular coverage and throughput (Park et al., 2022). These constraints mean that visually impressive maps are not automatically reproducible measurements. Practical adoption is also constrained by assay cost, long turnaround time, specialist instrumentation and bioinformatics staffing, limited tissue, reimbursement uncertainty, the scarcity of certified standardized kits and the absence of guideline-directed spatial-proteomic companion diagnostics for routine tumor treatment decisions.

Data interpretation is another challenge. Spatial proteomics can generate compelling tissue maps, but interpretation becomes descriptive if the study lacks a clear biological question. A robust spatial study should define tissue regions, cell types, marker panels and spatial metrics before analysis whenever possible. It should also distinguish exploratory findings from validated mechanisms. This distinction is especially important for small clinical cohorts, where high-dimensional spatial features can overfit outcomes.

Analytical rigor requires a null model matched to the biological question and tissue geometry rather than unrestricted randomization. Neighborhood analyses should preserve relevant cell-type abundance and boundaries, specify distance or graph scales *a priori*, correct for the number of spatial features tested and report sensitivity to alternative scales. Spatial autocorrelation must be modeled or tested because cells and pixels are not independent, and the patient, not each cell or ROI, should remain the unit of clinical inference. When multiple ROIs are sampled, patient-level aggregation or hierarchical models should account for within-patient dependence; performance should then be evaluated in held-out patients and, ideally, an external cohort (Samorodnitsky and Wu, 2024).

Standardization represents the most pressing need. To address this critical bottleneck in cross-study comparability, spatial proteomics studies should systematically report standardized information across at least seven core domains. This framework is explicitly aligned with the Minimum Information for Highly Multiplexed Tissue Images (MITI) guidelines (Schapiro et al., 2022) and the Human Tumor Atlas Network (HTAN) reporting conventions (Rozenblatt-Rosen et al., 2020), while extending them to include translational and clinical-modeling elements that are particularly relevant to cancer research. First, pre-analytic variables must be documented in detail, including tissue fixation, antigen retrieval, storage conditions, and section preparation methods. Second, platform and acquisition metadata should encompass instrument settings, scan parameters, batch information and records of any deviations during data collection. Third, antibody or probe identifiers and validation require provision of clone numbers, vendors, catalog numbers, working dilutions and validation status. Fourth, segmentation and phenotyping rules should be explicitly reported, detailing the algorithms used, input channels, parameter settings, manual correction workflows and logical gating strategies for cell-state assignment. Fifth, spatial-coordinate and neighborhood definitions must specify the graph construction method, neighborhood radius, distance metrics and the anatomical localization of regions of interest. Sixth, quality control thresholds and sensitivity analyses should cover signal intensity cutoffs, exclusion criteria for failed segmentation, and results of robustness tests against variations in key spatial parameters. Seventh, patient-level statistical models with prespecified endpoints must be clearly defined, ensuring that the patient remains the primary unit of clinical inference, with appropriate multiple-comparison correction strategies applied (Samorodnitsky and Wu, 2024). Adherence to the MITI and HTAN frameworks ensures interoperability with existing community resources such as the HTAN Data Portal and the Human Protein Atlas, while the additional translational elements address the specific needs of oncology studies seeking to move from tissue maps to clinically actionable biomarkers. SPIAT and related tools can help formalize certain aspects of these analyses, complete reporting in line with the framework above remains essential for meaningful cross-cohort comparison and eventual clinical translation.

A second challenge is biological validation. Spatial maps often nominate plausible interactions, but proximity does not prove signaling. A T cell near a tumor cell may be cytotoxic, exhausted, bystander or physically unable to engage. A fibroblast-rich region may be a barrier, a repair response or a marker of prior tissue damage. Spatial proteomics should therefore be paired with perturbation experiments, longitudinal sampling, orthogonal assays or clinical response data whenever possible. Treatment-linked breast cancer and melanoma immunotherapy studies illustrate why outcome-anchored designs are especially valuable.

A practical validation ladder begins with orthogonal confirmation of the nominated spatial feature by an independent assay on the same or adjacent tissue. Cell culture or co-culture experiments can then test directional signaling; patient-derived organoids or *ex vivo* tissue models can preserve multicellular context; genetically or pharmacologically perturbed animal models can test necessity, sufficiency and treatment response *in vivo*; and independent clinical cohorts with longitudinal sampling and therapeutic follow-up can establish prognostic or predictive relevance. Concordance across these levels is substantially stronger evidence than proximity alone.

The future of the field will likely depend on integration rather than platform competition. Spatial proteomics will be most powerful when combined with high-depth LC-MS/MS, spatial transcriptomics, single-cell sequencing, metabolomics and digital pathology. Spatial multi-omics reviews have framed this integration as a central route toward tissue-level systems biology. Clinical spatial omics guidelines also emphasize that integrated workflows must be standardized before broad clinical translation (Lee et al., 2025). AI-enabled virtual spatial proteomics may further increase scalability, but it requires rigorous external validation.

## 7 Conclusion

Spatial proteomics has shifted proteomics from averaged molecular catalogues toward tissue-resolved maps of biological function. In oncology, this transition is particularly important because tumor behavior is shaped not only by malignant-cell-intrinsic programs, but also by regional architecture, cellular neighborhoods and microenvironmental interactions. Current studies have shown that spatially resolved protein measurements can refine our understanding of intratumoral heterogeneity, tumor-immune organization, biomarker distribution and treatment-associated tissue states. These advances position spatial proteomics as an important bridge between molecular profiling, pathology and precision oncology.

Despite this progress, several barriers still limit broader translation. Spatial proteomic studies often differ in platform design, antibody panels, image processing, segmentation strategies and statistical workflows, making cross-cohort comparison difficult. Many findings also remain descriptive and require mechanistic validation in experimental models or prospective clinical cohorts. Future work should therefore move beyond higher multiplexing alone and emphasize standardized reporting, interoperable data analysis, clinically annotated samples and functional validation of spatially nominated targets. As these challenges are addressed, spatial proteomics is likely to become a central approach for interpreting tumor ecosystems and for linking tissue organization with clinically actionable cancer biology.
